# Association of Socioeconomic Position With Racial and Ethnic Disparities in Survival After Lung Transplant

**DOI:** 10.1001/jamanetworkopen.2023.8306

**Published:** 2023-04-19

**Authors:** Carli J. Lehr, Maryam Valapour, Paul R. Gunsalus, Warren T. McKinney, Kristen A. Berg, Johnie Rose, Jarrod E. Dalton

**Affiliations:** 1 Cleveland Clinic, Cleveland, Ohio; 2 Hennepin Healthcare Research Institute, Minneapolis, Minnesota; 3 The MetroHealth System, Case Western Reserve University, Cleveland, Ohio; 4 Case Western Reserve University, Cleveland, Ohio

## Abstract

**Importance:**

A recent National Academies of Sciences, Engineering, and Medicine study found that transplant outcomes varied greatly based on multiple factors, including race, ethnicity, and geographic location. They proposed a number of recommendations including studying opportunities to improve equity in organ allocation.

**Objective:**

To evaluate the role of donor and recipient socioeconomic position and region as a mediator of observed racial and ethnic differences in posttransplant survival.

**Design, Setting, and Participants:**

This cohort study included lung transplant donors and recipients with race and ethnicity information and a zip code tabulation area–defined area deprivation index (ADI) from September 1, 2011, to September 1, 2021, whose data were in the US transplant registry. Data were analyzed from June to December 2022.

**Exposures:**

Race, neighborhood disadvantage, and region of donors and recipients.

**Main Outcomes and Measures:**

Univariable and multivariable Cox proportional hazards regression were used to study the association of donor and recipient race with ADI on posttransplant survival. Kaplan-Meier method estimation was performed by donor and recipient ADI. Generalized linear models by race were fit, and mediation analysis was performed. Bayesian conditional autoregressive Poisson rate models (1, state-level spatial random effects; 2, model 1 with fixed effects for race and ethnicity, 3; model 2 excluding region; and 4: model 1 with fixed effects for US region) were used to characterize variation in posttransplant mortality and compared using ratios of mortality rates to the national average.

**Results:**

Overall, 19 504 lung transplant donors (median [IQR] age, 33 [23-46] years; 3117 [16.0%] Hispanic individuals, 3667 [18.8%] non-Hispanic Black individuals, and 11 935 [61.2%] non-Hispanic White individuals) and recipients (median [IQR] age, 60 [51-66] years; 1716 [8.8%] Hispanic individuals, 1861 [9.5%] non-Hispanic Black individuals, and 15 375 [78.8%] non-Hispanic White individuals) were included. ADI did not mediate the difference in posttransplant survival between non-Hispanic Black and non-Hispanic White recipients; it mediated only 4.1% of the survival difference between non-Hispanic Black and Hispanic recipients. Spatial analysis revealed the increased risk of posttransplant death among non-Hispanic Black recipients may be associated with region of residence.

**Conclusions and Relevance:**

In this cohort study of lung transplant donors and recipients, socioeconomic position and region of residence did not explain most of the difference in posttransplant outcomes among racial and ethnic groups, which may be due to the highly selected nature of the pretransplant population. Further research should evaluate other potentially mediating effects contributing to inequity in posttransplant survival.

## Key Points

**Question:**

Are racial disparities after lung transplant mediated by socioeconomic status and region?

**Findings:**

In this cohort study of 19 504 lung transplant donors and recipients, socioeconomic position and region did not explain most of the differences observed in posttransplant outcomes across race and ethnicity.

**Meaning:**

In this study, differential survival by race and ethnicity existed despite efforts to account for variation by socioeconomic position and region, a finding that highlights the presence of a complex social reality that warrants continued study to improve equity in posttransplant outcomes.

## Teaser

This cohort study evaluates the role of donor and recipient socioeconomic position and region as a mediator of observed racial and ethnic differences in post–lung transplant survival.

## Introduction

US Congress recently requested that the National Institutes of Health sponsor a National Academics of Sciences, Engineering, and Medicine study to review equity in the US organ transplant system. Marked variation and inefficiency in performance was found, with demonstratable inequity in the dimensions of race and ethnicity, geographic location, and socioeconomic position (SEP).^1^ In line with these findings, reports of US trends in survival after lung transplant have consistently shown differences across race and ethnicity.^2^ The lung allocation score is used to allocate organs to transplant candidates in the United States based on estimates of waitlist and posttransplant survival, but it excludes consideration of donor characteristics and recipient characteristics of race, SEP, and region as predictors despite their likely impact on posttransplant survival. Notably, race conveys 2 possible, related valences in the causal pathways as (1) a social classification with observable biological consequences that imprecisely map onto genetic or ancestral variation and (2) an indicator for likely exposure to systematic racism and implicit bias.^3^ There is a large body of literature that offers SEP as an explanation and alternative to presumptions that racial differences reflect group differences rooted in biological reality; however, the mediating role of SEP in racial disparities remains largely unexplored.

The Organ Procurement and Transplantation Network (OPTN) does not directly capture individual measures of SEP; however, recipient and donor location data allow estimation of SEP via neighborhood-level indicators such as the area deprivation index (ADI).^4^ The ADI is a measure of community-level socioeconomic disadvantage created by the Health Resources and Services Administration (HRSA) that includes domains of neighborhood economic hardship and inequality, financial strength, and educational attainment.^5,6^ A high ADI score reflects lower neighborhood resource availability or SEP and may reflect greater exposure to social risk, which can increase the number and strength of competing demands for time, reflecting an additional behavioral valence.^7^ When comparing the 4 major US census regions, differences emerge in age, racial composition, median income, self-reported health status, and comorbidities across populations.^8,9^ Regional disadvantage in access to kidney transplant exists, but data in thoracic transplant are limited.^10^ Geographic regions and neighborhood-level SEP measures may provide more granular data about donors and recipients in understanding the differences in survival associated with race and ethnicity. The objective of this study was to evaluate the extent to which racial and ethnic differences in posttransplant survival may be mediated by donor and recipient ADI and region.

## Methods

We identified lung organ donors and recipient pairs by match identifier from September 1, 2011, to September 1, 2021, using data from the Scientific Registry of Transplant Recipients (SRTR). The SRTR data system includes data on all donors, wait-listed candidates, and transplant recipients in the United States, submitted by the members of the OPTN. The HRSA, US Department of Health and Human Services, provides oversight to the activities of the OPTN and SRTR contractors. This study was approved by the Cleveland Clinic institutional review board. Informed consent was waived due to the presence of minimal risk and deidentified data. This study reported results according to the Strengthening the Reporting of Observational Studies in Epidemiology (STROBE) reporting guideline for cohort studies. ADI was assigned to the zip code tabulation area (ZCTA) of the permanent residence for all lung donors and recipients.^11^ SRTR collects clinically documented race and ethnicity classification groups including American Indian or Alaska Native, Asian or Pacific Islander, Hispanic, multiracial, non-Hispanic Black, or non-Hispanic White. Neighborhood SEP was operationalized by ADI score and evaluated as a continuous variable and by quintile (1, most resourced; 5, least resourced). Transplants by recipient race and ethnicity were compared by ADI quintile. US census regions included the Northeast, Midwest, South, and West regions.^12^

### Statistical Analysis

We compared posttransplant survival by donor and recipient ADI quintile using Kaplan-Meier and estimated associations between race and ethnicity, ADI, and posttransplant survival using Cox proportional hazards regression. We created univariable models for donor and recipient race and ethnicity followed by donor and recipient ADI, then estimated multivariable models combining donor race and ethnicity and ADI followed by recipient race and ethnicity and ADI. Models of race and ethnicity, ADI, and their interaction were estimated for both donors and recipients and compared using analysis of variance. Marginal effects were calculated to evaluate for racial and ethnic differences within ADI quintile.^13^ Recipient covariates were not adjusted for, as there was no mechanistic basis for consideration as potential confounders of donor effects on recipient survival. A Cox proportional hazard model including donor cause of death, HLA mismatch, blood type, and donor race and ethnicity was built to describe the extent to which racial and ethnic differences may be accounted for by other donor factors.

We used Poisson rate regression to compare posttransplant mortality rates and examine the extent to which these racial differences are accounted for by ADI. Pairwise comparisons were made between Hispanic, non-Hispanic Black, and non-Hispanic White recipients, with other racial categories excluded due to small sample sizes. Mediation analysis was used to build generalized linear models to quantify the natural direct and indirect (through ADI) effects of recipient race on posttransplant survival (eAppendix in Supplement 1).

We used a bayesian conditional autoregressive (CAR) Poisson rate model to characterize state-level variation in posttransplant mortality rates. This model directly estimated the ratio of deaths (numerator) to observable person-time (denominator) while accounting for correlation in outcomes among adjacent states. Observable person-time was calculated as the time between transplant and either death or date of last known follow-up. Four models were created: (1) a null model without fixed effects (included only state-level and spatial random effects); (2) a model with fixed effects for recipient and donor race and ethnicity, ADI, and region (ie, the full model); (3) a model with fixed effects for recipient and donor race and ethnicity and ADI, excluding region; and (4) a model with fixed effects for region only. The deviance information criterion (DIC) was used to compare models. Ratios of mortality rates comparing observed events to expected events (based on the national average of recipients over the cohort period) were calculated by state. Data analysis was conducted in R version 4.0.3 and Rstudio version 1.1.1093 (R Foundation for Statistical Computing). Statistical significance was set at *P* < .05, and tests were 2-tailed.

## Results

**Figure 1.**
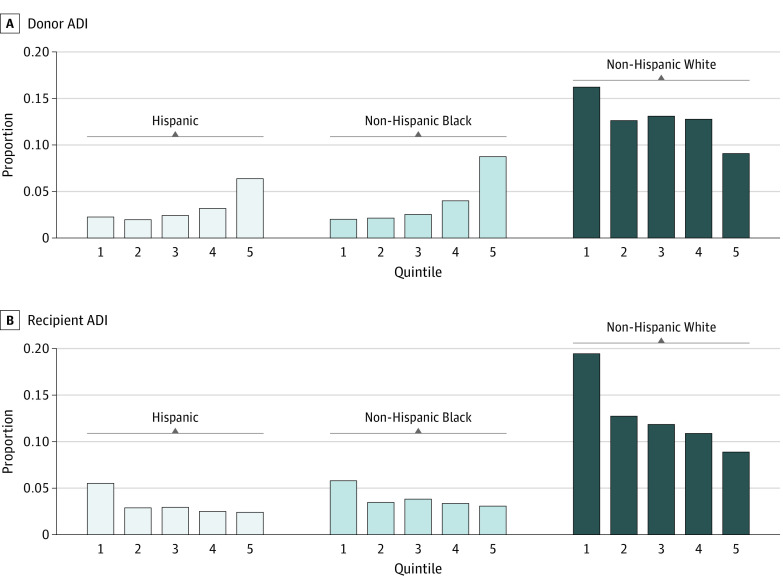
Grouped Bar Plots of Donor and Recipient Area Deprivation Index (ADI) Quintile by Race Proportion of donors within ADI quintile by race. Hispanic, non-Hispanic Black, and non-Hispanic White races and ethnicities are displayed given small sample sizes for American Indian or Alaska Native, Asian or Pacific Islander, and multiracial groups. ADI quintile 1 indicates the most resourced and 5, the least resourced.

A total of 19 504 lung transplant donor and recipient pairs were studied. The median (IQR) age of donors was 33 (23-46) years. Overall, 99 donors (0.5%) identified as American Indian or Alaska Native, 635 (3.2%) as Asian or Pacific Islander, 3117 (16.0%) as Hispanic, 51 (0.3%) as multiracial, 3667 (18.8%) as non-Hispanic Black, and 11 935 (61.2%) as non-Hispanic White; 4178 (21.4%) were classified in ADI quintile 1; 3255 (16.7%), quintile 2; 3506 (18.0%), quintile 3; 3868 (19.8%), quintile 4; and 4697 (24.1%), quintile 5. A total of 2506 donors (12.8%) resided in the Northeast; 8376 (42.9%), South; 4753 (24.4%), Midwest; and 3869 (19.8%), West. The median (IQR) age of recipients was 60 (51-66) years. Overall, 63 recipients (0.3%) identified as American Indian or Alaska Native, 442 (2.3%) as Asian or Pacific Islander, 1716 (8.8%) as Hispanic, 47 (0.2%) as multiracial, 1861 (9.5%) as non-Hispanic Black, and 15 375 (78.8%) as non-Hispanic White; 6122 (31.4%) were classified in ADI quintile 1; 3742 (19.2%), quintile 2; 3588 (18.4%), quintile 3; 3251 (16.7%), quintile 4; and 2801 (14.4%), quintile 5. A total of 3973 recipients (20.4%) resided in the Northeast; 7430 (38.1%), South; 4262 (21.9%), Midwest; and 3839 (19.7%), West (Table 1). Recipients identifying as non-Hispanic White had the highest proportion of individuals residing in the lowest ADI quintile (ie, most resourced) neighborhoods, while recipients identifying as non-Hispanic Black race and Hispanic ethnicity had the highest proportion of individuals residing in the highest ADI quintile (ie, least resourced) neighborhoods (Figure 1).

**Table 1.** Donor and Recipient Characteristics, Stratified by Recipient ADI Quintile

Characteristic	Recipients by ADI quintile, No. (%)^a^				
	1 (n = 6122)	2 (n = 3742)	3 (n = 3588)	4 (n = 3251)	5 (n = 2801)
Candidate age at listing, median (IQR), y	62 (52-67)	61 (53-66)	60 (51-65)	59 (51-65)	58 (49-64)
Race and ethnicity					
American Indian or Alaska Native	11 (0.2)	11 (0.3)	5 (0.1)	11 (0.3)	25 (0.9)
Asian or Pacific Islander	236 (3.9)	83 (2.2)	46 (1.3)	43 (1.3)	34 (1.2)
Hispanic	370 (6.0)	236 (6.3)	304 (8.5)	299 (9.2)	507 (18.1)
Multiracial	17 (0.3)	4 (0.1)	10 (0.3)	7 (0.2)	9 (0.3)
Non-Hispanic Black	321 (5.2)	274 (7.3)	301 (8.4)	339 (10.4)	626 (22.3)
Non-Hispanic White	5167 (84.4)	3134 (83.8)	2922 (81.4)	2552 (78.5)	1600 (57.1)
Diagnosis^b^					
A	1206 (19.7)	974 (26.0)	1009 (28.1)	956 (29.4)	738 (26.3)
B	270 (4.4)	159 (4.2)	146 (4.1)	140 (4.3)	147 (5.2)
C	612 (10)	345 (9.2)	333 (9.3)	304 (9.4)	214 (7.6)
D	4034 (65.9)	2264 (60.5)	2100 (58.5)	1851 (56.9)	1702 (60.8)
Blood type					
A	2429 (39.7)	1501 (40.1)	1433 (39.9)	1271 (39.1)	1022 (36.5)
AB	256 (4.2)	169 (4.5)	148 (4.1)	104 (3.2)	98 (3.5)
B	688 (11.2)	413 (11.0)	401 (11.2)	363 (11.2)	324 (11.6)
O	2749 (44.9)	1659 (44.3)	1606 (44.8)	1513 (46.5)	1357 (48.4)
BMI, median (IQR)	25.6 (21.8-29.1)	26.3 (22.3-29.3)	26.1 (22.1-29.2)	26.0 (22.1-29.2)	26.2 (22.4-29.5)
Unknown	3	1	1	1	3
Functional status					
No assistance	695 (11.4)	268 (7.2)	265 (7.4)	226 (7)	176 (6.3)
Some assistance	4975 (81.3)	3182 (85)	3050 (85)	2756 (84.8)	2360 (84.3)
Full assistance	438 (7.2)	283 (7.6)	266 (7.4)	254 (7.8)	250 (8.9)
Unknown	14	9	7	15	15
Six-min walk distance, median (IQR), m	850 (500-1128)	843 (535-1100)	850 (525-1110)	820 (492-1100)	800 (456-1070)
Unknown	114	68	57	73	49
Cardiac Index, median (IQR)	2.80 (2.40-3.30)	2.78 (2.40-3.20)	2.80 (2.41-3.26)	2.80 (2.40-3.28)	2.80 (2.40-3.32)
Unknown	1584	963	924	836	754
Bilirubin, median (IQR), mg/dL	0.50 (0.30-0.70)	0.50 (0.30-0.60)	0.45 (0.30-0.70)	0.50 (0.30-0.60)	0.40 (0.30-0.60)
Unknown	1879	1192	1080	1034	794
Creatinine, median (IQR), mg/dL	0.80 (0.69-0.97)	0.80 (0.69-1.00)	0.80 (0.69-0.96)	0.80 (0.68-0.97)	0.80 (0.67-0.96)
Unknown	24	15	18	11	17
Supplemental oxygen, median (IQR), L	4.0 (3.0-6.0)	4.0 (3.0-6.0)	4.0 (3.0-6.0)	4.0 (2.5-6.0)	4.0 (3.0-6.0)
Unknown	801	505	497	453	410
Respiratory support					
BiPAP	390 (6.4)	240 (6.4)	276 (7.7)	276 (8.5)	214 (7.6)
Mechanical	353 (5.8)	203 (5.4)	194 (5.4)	183 (5.6)	167 (6)
None	5014 (81.9)	3070 (82.0)	2913 (81.2)	2611 (80.3)	2262 (80.8)
CPAP	356 (5.8)	222 (5.9)	199 (5.5)	175 (5.4)	153 (5.5)
Unknown	9	7	6	6	5
Pco_2_, median (IQR), mm Hg	44 (38-51)	44 (39-52)	45 (39-53)	45 (39-53)	45 (39-52)
Unknown	260	169	172	131	117
Supplemental oxygen, median (IQR), %	100 (56-100)	90 (57-100)	80 (52-100)	84 (60-100)	90 (52-100)
Unknown	5495	3330	3194	2867	2471
Pulmonary artery systolic pressure, median (IQR), mm Hg	38 (31-48)	39 (32-49)	39 (32-48)	39 (32-49)	40 (33-50)
Unknown	1382	807	785	719	643
Donor age, median (IQR), y	33 (23-47)	33 (23-47)	33 (23-46)	33 (23-46)	32 (22-46)
Donor race					
American Indian or Alaska Native	33 (0.5)	14 (0.4)	16 (0.4)	20 (0.6)	16 (0.6)
Asian or Pacific Islander	209 (3.4)	140 (3.7)	108 (3.0)	102 (3.1)	76 (2.7)
Hispanic	1078 (17.6)	547 (14.6)	556 (15.5)	476 (14.6)	460 (16.4)
Multiracial	23 (0.4)	11 (0.3)	6 (0.2)	5 (0.2)	6 (0.2)
Non-Hispanic Black	1093 (17.9)	659 (17.6)	710 (19.8)	628 (19.3)	577 (20.6)
Non-Hispanic White	3686 (60.2)	2371 (63.4)	2192 (61.1)	2020 (62.1)	1666 (59.5)
Donor ADI quintile					
1	1485 (24.3)	828 (22.1)	688 (19.2)	632 (19.4)	545 (19.5)
2	1015 (16.6)	660 (17.6)	611 (17)	544 (16.7)	425 (15.2)
3	1050 (17.2)	705 (18.8)	665 (18.5)	591 (18.2)	495 (17.7)
4	1141 (18.6)	707 (18.9)	762 (21.2)	683 (21.0)	575 (20.5)
5	1431 (23.4)	842 (22.5)	862 (24)	801 (24.6)	761 (27.2)

Abbreviations: ADI, area deprivation index; BiPAP, bilevel positive airway pressure; BMI, body mass index (calculated as weight in kilograms divided by height in meters squared); CPAP, continuous positive airway pressure; Pco_2_, partial pressure of carbon dioxide.

SI conversion factors: To convert bilirubin to micromoles per liter, multiply by 17.104; creatinine to micromoles per liter, multiply by 88.4.

^a^ ADI quintile 1 indicates most resourced and 5, least resourced.

^b^ Diagnosis group A indicates obstructive lung disease; group B, pulmonary vascular disease; group C, cystic fibrosis and immunodeficiency disorders; and group D, restrictive lung diseases.

### Donor Race and ADI

In the univariable Cox proportional hazards model, receipt of an organ from a non-Hispanic Black donor was associated with a 23% increased risk of posttransplant mortality (hazard ratio [HR], 1.23 [95% CI, 1.16-1.31]; *P* < .001). Receipt of an organ from a donor residing in a neighborhood in the highest ADI quintile was associated with a 14% increased risk of posttransplant mortality (HR, 1.14 [95% CI, 1.06-1.22]; *P* < .001). In the multivariable model accounting for donor race, ethnicity, and ADI, the association between receiving an organ from a non-Hispanic Black donor and increased risk of posttransplant mortality was not attenuated, suggesting that this association was not mediated through ADI. The association of ADI remained but was attenuated from a 14% increased risk in the univariate model to an 8% increased risk in the multivariable model after adjusting for race and ethnicity (Table 2). The model assuming ADI as an effect modifier of racial disparities (interaction model) was significantly different from the main model used in mediation analysis (*P* = .02). However, estimates of non-Hispanic White compared with non-Hispanic Black racial differences were consistent across the 5 ADI quintiles (eTable 1 in Supplement 1). Donor cause of death, blood type, and HLA mismatch were not associated with posttransplant survival, suggesting that the observed racial and ethnic differences may be driven by other social or biological mechanisms (eTable 2 in Supplement 1).

**Table 2.** Cox Proportional Hazards Model: Posttransplant Survival by Donor and Recipient Race and Ethnicity and ADI^a^

Characteristic	Race		ADI		Race and ADI	
	HR (95% CI)	*P* value	HR (95% CI)	*P* value	HR (95% CI)	*P* value
**Donor**						
Race and ethnicity						
American Indian or Alaska Native	1.16 (0.83-1.60)	.40	NA	NA	1.14 (0.82-1.59)	.40
Asian or Pacific Islander	1.13 (0.99-1.29)	.08	NA	NA	1.14 (0.99-1.30)	.06
Hispanic	1.07 (1.00-1.14)	.06	NA	NA	1.05 (0.98-1.13)	.14
Multi-racial	1.14 (0.68-1.93)	.60	NA	NA	1.13 (0.67-1.91)	.70
Non-Hispanic Black	1.23 (1.16-1.31)	<.001	NA	NA	1.22 (1.14-1.29)	<.001
Non-Hispanic White	1 [Reference]	NA	NA	NA	1 [Reference]	NA
ADI quintile						
1	NA	NA	1 [Reference]	NA	1 [Reference]	NA
2	NA	NA	1.07 (0.99-1.16)	.09	1.07 (0.99-1.15)	.11
3	NA	NA	1.04 (0.96-1.12)	.40	1.03 (0.95-1.11)	.50
4	NA	NA	1.03 (0.96-1.11)	.40	1.01 (0.94-1.09)	.80
5	NA	NA	1.14 (1.06-1.22)	<.001	1.08 (1.00-1.16)	.049
**Recipient**						
Race and ethnicity						
American Indian or Alaska Native	0.97 (0.61-1.54)	.90	NA	NA	0.97 (0.61-1.54)	.90
Asian or Pacific Islander	0.92 (0.77-1.10)	.40	NA	NA	0.91 (0.76-1.09)	.30
Hispanic	0.94 (0.86-1.03)	.20	NA	NA	0.94 (0.86-1.03)	.20
Multi-racial	1.11 (0.68-1.81)	.70	NA	NA	1.10 (0.68-1.80)	.70
Non-Hispanic Black	1.11 (1.02-1.20)	.01	NA	NA	1.10 (1.02-1.20)	.02
Non-Hispanic White	1 [Reference]	NA	NA	NA	1 [Reference]	NA
ADI quintile						
1	NA	NA	1 [Reference]	NA	1 [Reference]	NA
2	NA	NA	0.96 (0.89-1.03)	.20	0.95 (0.89-1.02)	.20
3	NA	NA	0.92 (0.85-0.98)	.02	0.91 (0.85-0.98)	.01
4	NA	NA	1.01 (0.94-1.08)	.80	1.00 (0.93-1.08)	>.99
5	NA	NA	0.99 (0.92-1.07)	.90	0.98 (0.91-1.06)	.60

Abbreviations: ADI, area deprivation index; HR, hazard ratio; NA, not applicable.

^a^ The donor race model is a univariable model and adjusted for donor race alone. Donor ADI is a univariable model and adjusted for donor ADI alone. Donor race and ADI model is a multivariable variable and adjusted for both donor race and ADI. Recipient race model is a univariable model and adjusted for recipient race alone. Recipient ADI is a univariable model and adjusted for recipient ADI alone. Recipient race and ADI model is a multivariable model and adjusted for both recipient race and ADI. Attenuation of the hazard ratio indicates potential confounding.

**Figure 2.**
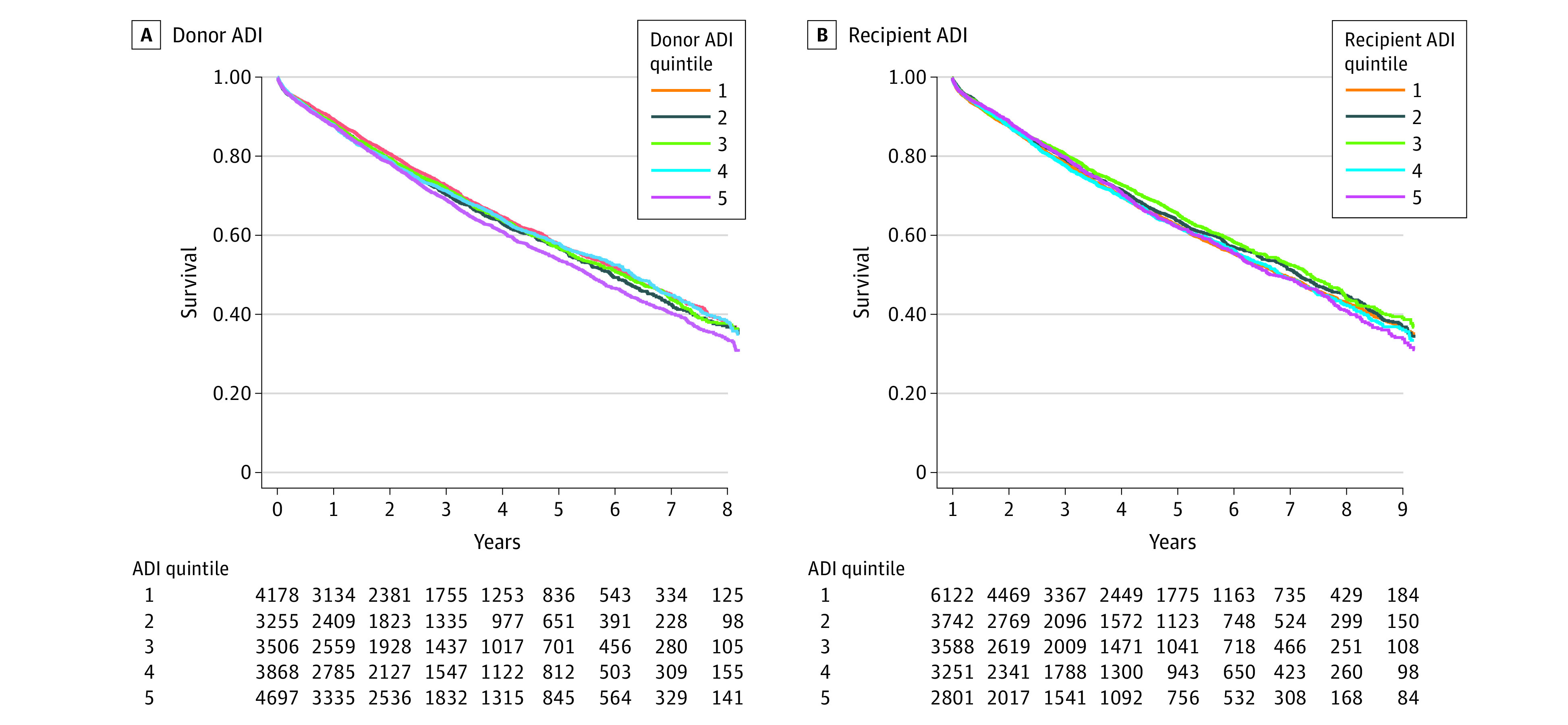
Kaplan-Meier Survival Models by Area Deprivation Index (ADI) Donor and Recipient Quintiles

**Figure 3.**
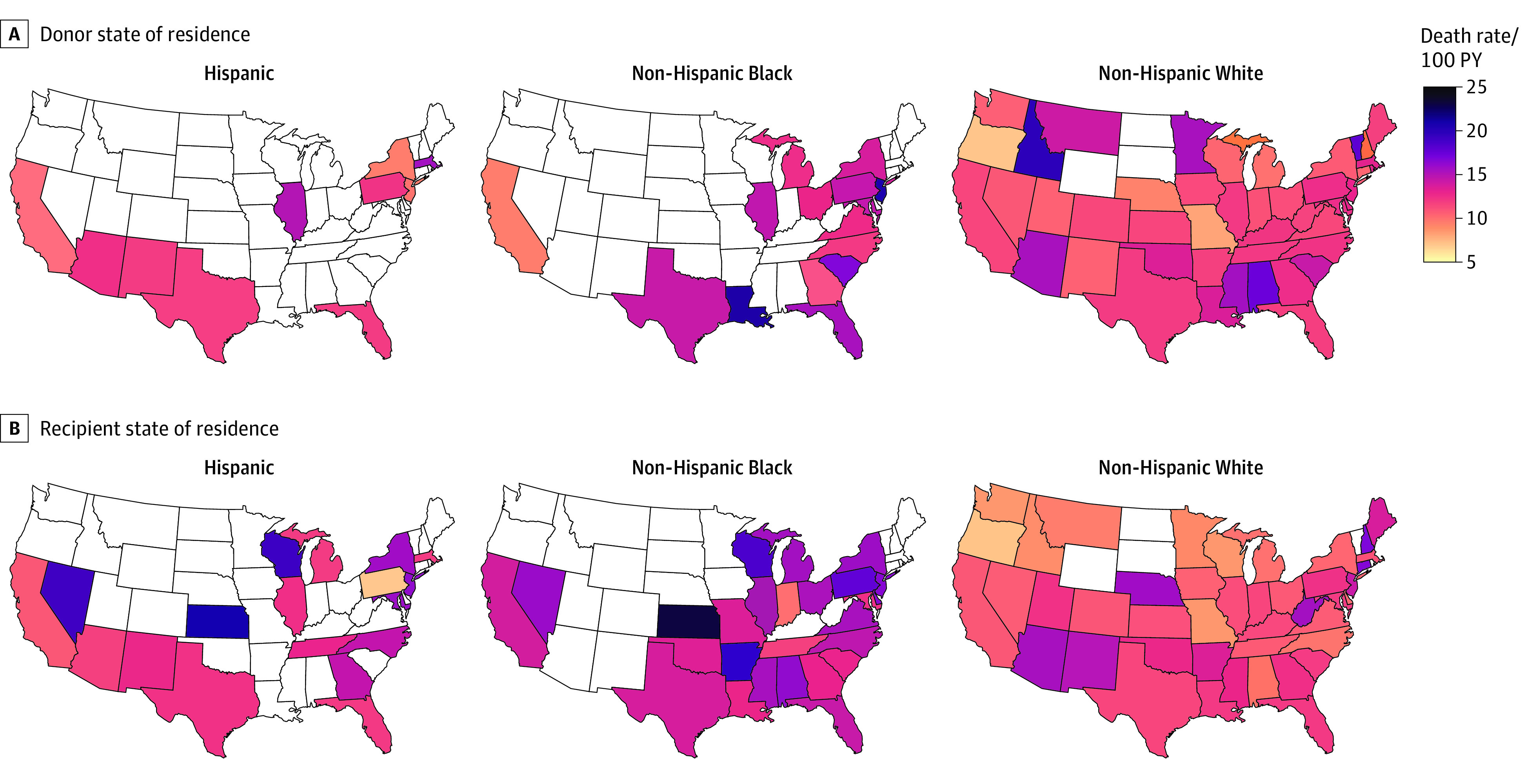
Posttransplant Recipient Death Rates by Donor and Recipient State of Residence Maps presented by donor (A) and recipient (B) race and ethnicity. Deaths represented as rates per 100 person-years (PY). Data are suppressed for states with 10 or fewer donors (A) or recipients (B).

Kaplan-Meier analysis indicated worse survival for recipients who received an organ from a donor residing in neighborhoods in the highest ADI quintile (*P* = .003) (Figure 2A). Observed mortality rates differed by donor racial and ethnic groups (Figure 3A). There were generally higher rates of death for recipients with non-Hispanic Black donors compared with those with non-Hispanic White donors. Hispanic donor trends closely resembled those seen for non-Hispanic Black donors (Figure 3A).

### Recipient Race and ADI

In univariate models, non-Hispanic Black (vs non-Hispanic White) recipients experienced an 11% increased risk of posttransplant mortality (HR, 1.11 [95% CI, 1.02-1.20]; *P* = .01). This association was not attenuated in multivariable modeling (Table 2). Recipients residing in neighborhoods in the third ADI quintile experienced a slightly reduced risk of posttransplant mortality (HR, 0.92 [95% CI, 0.85-0.98]; *P* = .02), but this is likely the result of statistical uncertainty and unlikely to be reflective of a larger trend. The model accounting for interactions was not significant (*P* = .86), and the only marginal effect that was significant was comparing non-Hispanic White and non-Hispanic Black recipients within ADI quintile 5 (eTable 3 in Supplement 1).

Posttransplant mortality rates for non-Hispanic Black recipients were 18% (95% CI, 3%-36%) higher than those for Hispanic recipients and 11% (95% CI, 1%-22%) higher than those for non-Hispanic White recipients. The difference in posttransplant mortality between non-Hispanic White and Hispanic recipients was not statistically significant. In mediation analysis comparing non-Hispanic Black recipients and Hispanic recipients, only 4.1% of the association of race was mediated by ADI (*P* = .006). We did not find evidence of a mediating effect of neighborhood socioeconomic deprivation on the difference in posttransplant mortality rates between non-Hispanic Black and non-Hispanic White recipients.

Kaplan-Meier analysis demonstrated similar survival across recipient ADI quintiles (*P* = .09) (Figure 2B). Mortality rates varied across recipient racial and ethnic groups (Figure 3B). Non-Hispanic Black recipients experienced a higher risk of death compared with non-Hispanic White or Hispanic recipients in most states with enough data to report (ie, ≥10 deaths) (Figure 3B). Ratios of mortality rates compared with the national average by recipient state of residence ranged from 0.54 to 1.69 (eFigure and eTable 4 in Supplement 1).

### Spatial Analysis

The full CAR model showed an increased risk for posttransplant mortality for recipients who received an organ from a donor residing in the least-resourced neighborhoods and who were of non-Hispanic Black or Asian or Pacific Islander race. Regional associations were observed, with an increased risk of posttransplant mortality for recipients who received organs from the Northeast in the full CAR model. However, the CAR model that used fixed effects for region alone found recipients from the South were at a greater risk for poor posttransplant outcomes. Associations with non-Hispanic Black donor race and the highest quintile of donor ADI persisted, while non-Hispanic Black recipient race emerged as a risk factor when donor and recipient region were removed from the model, suggesting the increased risk of posttransplant death among non-Hispanic Black recipients may be related to region. Models performed similarly by DIC (eTable 5 in Supplement 1).

## Discussion

### Principal Findings

We found that non-Hispanic Black recipients of lung transplants and recipients of non-Hispanic Black donors experienced lower posttransplant survival. Controlling for ADI, a multidimensional index capturing neighborhood socioeconomic position that has been strongly linked to health outcomes, failed to ameliorate the observed associations with race.

### Racial Differences and ADI

We found that neighborhood SEP, as measured by ADI, did not account for racial and ethnic disparities in survival after lung transplant. There are a number of proposed mechanisms to explain why racial and ethnic variation in posttransplant survival was not mediated by ADI or region. First, as a singular index, ADI cannot fully capture the diversity of neighborhood environments and corresponding social and economic risks affecting lung health, access to care, and an individual’s ability to successfully maintain complex posttransplant medical care. Additionally, the socioeconomic indicators used in the ADI are neither temporally nor spatially invariant and may reflect subdimensions of poverty and disadvantage that are insufficiently described by a single index.^6^ Moreover, data constraints limited operationalization of neighborhood as ZCTAs rather than as more precise, granular small-area units, such as the census tract or block group. Given measurement error inherent in the geographic imprecision of ZCTAs as well as their boundary instability and incomplete geographic coverage analyses may have underestimated any intervening effect of neighborhood SEP as measured by the ADI.

Second, biological embodiment of social adversity and associated injustices that occur across the life course of racially minoritized individuals may contribute to outcomes.^14,15^ Numerous studies have indicated heightened inflammatory status and reduced immune function among those with childhood histories of trauma and adversity, and it is plausible that organ quality and lung function later in the life course may be secondarily impacted by these mechanisms.^16,17^ Given that racial designations are not biological concepts but, rather, have biological consequences given structures of inequality that reciprocally reinforce race-based health disparities, there have been efforts to understand biological and epigenetic mechanisms underpinning racial differences in thoracic transplant.^18,19^ Investigators have found higher incidence of rejection, graft dysfunction, and death in non-Hispanic Black heart transplant recipients.^20^ Higher levels of donor-derived cell-free DNA have been isolated in Black heart transplant recipients, a finding that may be mediated by donor and recipient race mismatch, which is more common for Black recipients.^19^ Multiple studies have shown improved survival in matched-race thoracic transplant.^21,22^

Third, a social ecological model with domains affecting posttransplant care with domains of intrapersonal, interpersonal, institutional, community, and public policy processes, impacted by race and/or racism, may further explain differential survival outcomes.^23,24,25^ Studies have come to varying conclusions regarding posttransplant medical care and compliance with medication regimens.^26,27,28^

Finally, neighborhood-level area deprivation may not align well with exposure to structural racism and everyday experiences of discrimination and implicit bias. In particular, the ADI lacks explicit equity variables that track outcomes in a comparative framework that considers patterned distributions. Studies using a novel ecological-level multidimensional measures of structural racism found that non-Hispanic Black–non-Hispanic White segregation and inequities in education, employment, income, and homeownership mediate disparities in birth rates and vaccination rates.^29^

### Association of ADI With Survival Independent of Race

While much of the association of race and ethnicity with posttransplant outcomes was not mediated by ADI, a significant ADI association independent from those seen with race and ethnicity existed for recipients who were matched with donors from the highest ADI quintile. We hypothesize this association may be explained by the effects of neighborhood environment and reflect risk that is independent of race and ethnicity within the population. The mediation of ADI may be stronger and more easily detected in disciplines such as preventative medicine due to a survivorship effect in which individuals may appear to have lower risk due to competing risks of death, as individuals die from other causes before becoming at-risk for the outcome.^30^

Geographic differences in health and mortality exist in the United States with clear differences at multiple levels of spatial resolution.^9,31,32^ Regional disparities in access to transplant have been the primary driver leading to major restructuring of US organ allocation systems, leading us to study regional variation independent of race and ethnicity and ADI in this work.^33^ For example, an increased mortality risk of 7% existed for recipients residing in the South in the region-only model, but this was attenuated when accounting for race and ethnicity and ADI. However, the effect size for Northeast donors remained stable despite adjustment. Further analysis of these regional differences with higher degrees of spatial granularity—to the extent that available data become sufficiently large for reliable modeling—are required to understand the complexity of these associations.

As US lung allocation moves to adopt the Composite Allocation Score system, the impact of posttransplant survival may introduce or exacerbate potential race- and ethnicity-based disparities in allocation. Current allocation models do not explicitly include race or ethnicity to prevent introduction of potential bias into allocation.^34^ Exclusion of race in existing survival models prohibits complete understanding of contributions of minority racial or ethnic status to posttransplant survival, and deleterious effects may be exacerbated with the increased emphasis on this outcome. It is possible that unmeasured consequences of race and ethnicity may be seen through other clinical risk factors, including panel-reactive antibodies, blood type, pulmonary function, and/or exposure to structural racism.

### Limitations

This study has limitations, including the reliance on a national transplant database that does not collect granular measures of residential location or individualized SEP data. Our use of a ZCTA-level ADI measure, as was necessary given data limitations, may have led to conclusions that are vulnerable to ecologic fallacy, where it is assumed that a group-level attribute is reflective of individuals in that group. Our mediation findings are based on proportional hazards assumptions over time, which are common in survival analysis, but may not be reflective of causality to the extent that the structure of the transplant recipient population changes as a function of time.^35^

## Conclusions

In this study, we found that the observed racial and ethnic differences in posttransplant survival among lung transplant recipients were not fully explained by neighborhood SEP and region. This is likely due to the highly selected nature of the population, measurement imprecision in assessing neighborhood SEP, and a clinically homogenous residual cohort more insulated from social and economic stressors compared with the end-stage lung disease population. Future research should evaluate mediating effects of pulmonary and other chronic comorbidities on disparities and compare these with those of other environmental factors not adequately captured by the ADI score. Many of the adverse effects of structural racism^36^ described and socioeconomic disadvantage are upstream of the point of accessing the transplant waiting list, broadly impact multiple biologic systems, and exert differential mortality pressures that exclude vulnerable groups from the population of eligible lung transplant candidates. In the context of this study, it was found that neighborhood SEP did not mediate the associations of race and ethnicity with survival, but it had its own association. This reflects a far more complex social reality than is often presented in health services research.^37,38^

## Notes

Supplement 1. **eAppendix.** Mediation Analysis Methods **eTable 1.** Marginal Effects of Donor Race and Ethnicity Across ADI Quintile **eTable 2.** Cox Proportional Hazards Model by Donor Factors **eTable 3.** Marginal Effects of Recipient Race and Ethnicity Across ADI Quintile **eFigure.** Ratios of Mortality Rates by State **eTable 4.** Standardized Mortality Ratios By State Across Bayesian Models **eTable 5.** Donor and Recipient Characteristics by US Census Region
eReferences.

Supplement 2.
Data Sharing Statement
