# Supplementary material for: Association of Socioeconomic Position With Racial and Ethnic Disparities in Survival After Lung Transplant
Source: JAMA Netw Open. 2023 Apr 19;6(4):e238306. doi: 10.1001/jamanetworkopen.2023.8306 (PMC10116361; doi:10.1001/jamanetworkopen.2023.8306)
Supplement: Supplement 1. — eAppendix. Mediation Analysis Methods eTable 1. Marginal Effects of Donor Race and Ethnicity Across ADI Quintile eTable 2. Cox Proportional Hazards Model by Donor Factors eTable 3. Marginal Effects of Recipient Race and Ethnicity Across ADI Quintile eFigure. Ratios of Mortality Rates by State eTable 4. Standardized Mortality Ratios By State Across Bayesian Models eTable 5. Donor and Recipient Characteristics by US Census Region eReferences. [file jamanetwopen-e238306-s001.pdf]

Supplemental Online Content

Lehr CJ, Valapour M, Gunsalus PR, et al. Association of socioeconomic position with racial and ethnic disparities in survival after lung transplant. *JAMA Netw Open.* 2023;6(4):e238306. doi:10.1001/jamanetworkopen.2023.8306

eAppendix. Mediation Analysis Methods

eTable 1. Marginal Effects of Donor Race and Ethnicity Across ADI Quintile

eTable 2. Cox Proportional Hazards Model by Donor Factors

eTable 3. Marginal Effects of Recipient Race and Ethnicity Across ADI Quintile

eFigure. Ratios of Mortality Rates by State

eTable 4. Standardized Mortality Ratios By State Across Bayesian Models

eTable 5. Donor and Recipient Characteristics by US Census Region

eReferences.

This supplemental material has been provided by the authors to give readers additional information about their work.

© 2023 Lehr CJ et al. *JAMA Network Open.*

eAppendix. Mediation Analysis Methods

Mediation analysis was used to build generalized linear models to quantify the natural direct and indirect (through ADI) effects of recipient race on post-transplant survival. Estimation of the natural indirect effect was reasonable given that the following four assumptions were met: first, there was no unmeasured confounding in the relationship between recipient race and the post-transplant survival outcome (X and Y, respectively, in the X-M-Y model). Second, there was no unmeasured confounding of the relationship between neighborhood socioeconomic position (M) and the post-transplant survival outcome (Y) and nor was there, third, unmeasured confounding between race (X) and neighborhood socioeconomic position (M). Lastly, there was no observed or unobserved confounding between neighborhood socioeconomic position (M) and the post-transplant survival outcome (Y) that is caused by race (X)[1]. The prior-described interaction models of race and ADI evaluate the alternative possibility that neighborhood socioeconomic position modifies the relationship between race and post-transplant survival. A directed acyclic graph is presented in the appendix.

Directed Acyclic Graph Guiding Study Design

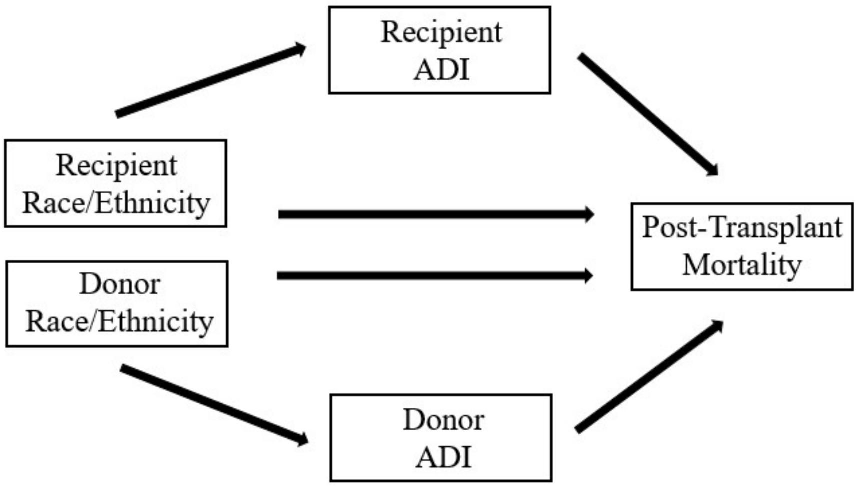

© 2023 Lehr CJ et al. *JAMA Network Open.*

eTable 1. Marginal Effects of Donor Race and Ethnicity Across ADI Quintile

Tukey method for comparing a family of 5 estimates was used.

Comparison	Donor ADI Quintile	Estimate	Z Ratio	p-value
NHW - Hispanic	1	0.877	-1.501	0.561
NHW-NHB	1	0.740	-3.529	0.003
NHW - Asian/Pacific Islander	1	0.842	-1.639	0.472
NHW - Native American	1	1.091	0.123	0.999
NHW - Hispanic	2	1.063	0.619	0.972
NHW - NHB	2	0.868	-1.614	0.488
NHW - Asian/Pacific Islander	2	0.814	-1.178	0.764
NHW - Native American	2	1.829	1.043	0.835
NHW - Hispanic	3	0.942	-0.673	0.962
NHW - NHB	3	0.907	-1.220	0.740
NHW - Asian/Pacific Islander	3	1.414	1.640	0.472
NHW - Native American	3	3.431	1.742	0.408
NHW - Hispanic	4	0.944	-0.725	0.951
NHW - NHB	4	0.771	-3.842	0.001
NHW - Asian/Pacific Islander	4	0.853	-0.840	0.918
NHW - Native American	4	0.382	-3.925	0.001
NHW - Hispanic	5	0.942	-0.920	0.889
NHW - NHB	5	0.829	-3.267	0.010
NHW - Asian/Pacific Islander	5	0.723	-1.981	0.275
NHW - Native American	5	0.883	-0.425	0.993

© 2023 Lehr CJ et al. *JAMA Network Open.*

eTable 2. Cox Proportional Hazards Model by Donor Factors

Characteristic	HR (95% CI)[1]	p-value
Donor Race		
Non-Hispanic White	—	
Hispanic	1.06 (0.98, 1.14)	0.13
Non-Hispanic Black	1.22 (1.14, 1.30)	<0.001
Asian/Pacific Islander	1.09 (0.95, 1.26)	0.2
Multi-Racial	1.14 (0.66, 1.97)	0.6
Native American	1.11 (0.80, 1.55)	0.5
Cause of Death		
Anoxia	—	
CVA	1.04 (0.97, 1.11)	0.2
Head Trauma	0.96 (0.90, 1.02)	0.2
CNS Tumor	0.82 (0.56, 1.20)	0.3
Other	1 (0.85, 1.18)	>0.9
Blood Type		
A	—	
AB	1.03 (0.87, 1.21)	0.8
B	0.96 (0.88, 1.04)	0.3
O	1.02 (0.97, 1.08)	0.4
HLA Mismatches		
0	—	
1	0.98 (0.38, 2.51)	>0.9
2	1.2 (0.49, 2.91)	0.7
3	1.2 (0.50, 2.89)	0.7
4	1.13 (0.47, 2.72)	0.8
5	1.23 (0.51, 2.96)	0.6
6	1.34 (0.55, 3.21)	0.5

[1] HR = Hazard Ratio, CI = Confidence Interval

© 2023 Lehr CJ et al. *JAMA Network Open.*

eTable 3. Marginal Effects of Recipient Race and Ethnicity Across ADI Quintile

Tukey method for comparing a family of 5 estimates was used.

Comparison	Recipient ADI Quintile	Estimate	Z Ratio	p-value
NHW - Hispanic	1	1.169	1.539	0.639
NHW - NHB	1	0.984	-0.164	0.999
NHW - Asian/Pacific Islander	1	1.137	1.038	0.905
NHW - Multi-Racial	1	1.385	0.651	0.987
NHW - Native American	1	1.097	0.161	0.999
NHW - Hispanic	2	1.118	0.914	0.943
NHW - NHB	2	0.920	-0.790	0.969
NHW - Asian/Pacific Islander	2	1.236	0.984	0.923
NHW - Multi-Racial	2	0.620	-0.675	0.985
NHW - Native American	2	0.737	-0.529	0.995
NHW - Hispanic	3	1.121	0.955	0.932
NHW - NHB	3	0.887	-1.163	0.854
NHW - Asian/Pacific Islander	3	1.401	0.949	0.933
NHW - Multi-Racial	3	0.857	-0.267	0.999
NHW - Native American	3	2.193	0.785	0.970
NHW - Hispanic	4	1.034	0.299	0.999
NHW - NHB	4	0.984	-0.168	0.999
NHW - Asian/Pacific Islander	4	0.976	-0.086	0.999
NHW - Multi-Racial	4	0.755	-0.398	0.999
NHW - Native American	4	1.117	0.192	0.999
NHW - Hispanic	5	0.916	-0.965	0.929
NHW - NHB	5	0.789	-3.042	0.028
NHW - Asian/Pacific Islander	5	0.626	-1.604	0.596
NHW - Multi-Racial	5	0.696	-0.806	0.967
NHW - Native American	5	0.897	-0.305	0.999

© 2023 Lehr CJ et al. *JAMA Network Open.*

eFigure 1. Ratios of Mortality Rates by State

Ratios of mortality rates calculated by dividing the observed number of deaths per state by the corresponding model predicted per state using the Bayesian conditional autoregressive (CAR) Poisson rate model.

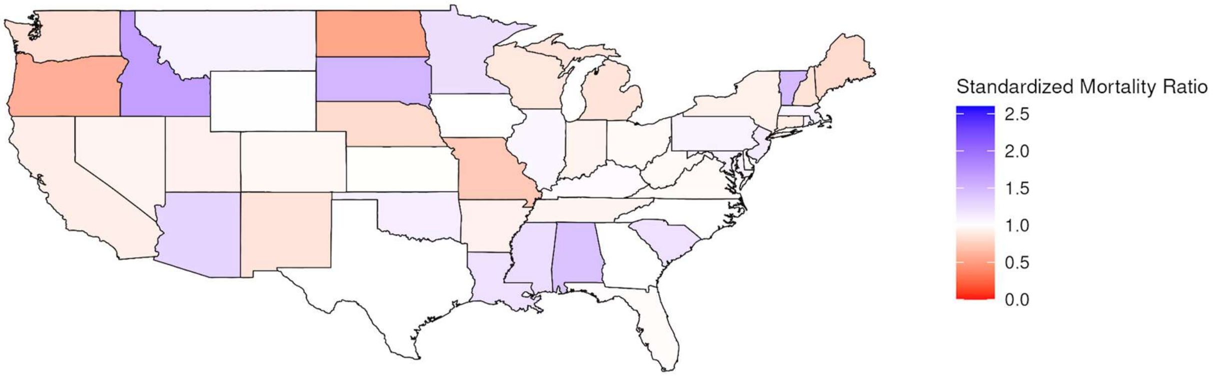

© 2023 Lehr CJ et al. *JAMA Network Open.*

eTable 4. Standardized Mortality Ratios By State Across Bayesian Models
Model 1) Spatial autocorrelation alone, Model 2) Spatial autocorrelation with candidate and donor race, ADI, and US census region, Model 3) Spatial autocorrelation with candidate and donor race, ADI, excluding US census region, and Model 4) Spatial autocorrelation and region alone. Model 1 SMRs were mapped in Figure 4.

SMR, Standardized Mortality Ratio

Recipient State	Total Deaths	Survival Time	Model 1 SMR	Model 2 SMR	Model 3 SMR	Model 4 SMR
AL	101	215281	1.41	1.34	1.34	1.37
AR	45	148845	0.93	0.87	0.90	0.90
AZ	290	686208	1.30	1.33	1.32	1.32
CA	617	2065799	0.91	0.94	0.92	0.94
CO	95	302742	0.96	0.98	0.97	0.97
CT	61	206059	0.90	0.87	0.88	0.89
DE	39	123045	0.96	0.88	0.94	0.90
FL	418	1242098	0.99	0.98	0.99	0.95
GA	146	440631	1.00	0.97	0.97	0.98
IA	31	93259	1.02	1.11	1.05	1.11
ID	26	47612	1.65	1.70	1.73	1.60
IL	288	838132	1.05	1.11	1.04	1.13
IN	208	682013	0.94	1.01	0.96	1.01
KS	53	159573	0.99	1.06	1.02	1.04
KY	137	409779	1.02	1.00	1.02	1.01
LA	133	335113	1.20	1.11	1.15	1.15
MA	144	404171	1.08	1.05	1.10	1.03
MD	181	507862	1.09	1.03	1.05	1.05
ME	24	79448	0.81	0.78	0.84	0.77
MI	237	845991	0.86	0.92	0.87	0.93
MN	40	94452	1.22	1.30	1.24	1.25
MO	102	436761	0.72	0.78	0.73	0.77
MS	54	130706	1.23	1.18	1.19	1.18
MT	20	50812	1.09	1.12	1.13	1.10
NC	240	724667	1.00	0.97	0.98	0.97
ND	5	27106	0.54	0.60	0.57	0.54

© 2023 Lehr CJ et al. *JAMA Network Open.*

Recipient State	Total Deaths	Survival Time	Model 1 SMR	Model 2 SMR	Model 3 SMR	Model 4 SMR
NE	24	92006	0.81	0.89	0.83	0.87
NH	27	95233	0.85	0.81	0.86	0.81
NJ	249	669932	1.14	1.07	1.11	1.09
NM	43	153847	0.86	0.88	0.89	0.89
NV	36	119724	0.93	0.97	0.96	0.95
NY	314	1044297	0.93	0.90	0.92	0.91
OH	333	1057029	0.97	1.02	0.97	1.03
OK	95	260988	1.11	1.09	1.12	1.08
OR	32	173411	0.58	0.62	0.60	0.60
PA	522	1482880	1.08	1.05	1.08	1.06
RI	15	41745	1.07	1.07	1.07	1.05
SC	89	221355	1.20	1.15	1.18	1.14
SD	11	20286	1.50	1.68	1.59	1.69
TN	105	337134	0.95	0.94	0.95	0.92
TX	717	2146904	1.00	0.97	1.00	0.97
UT	18	59382	0.93	1.00	0.99	0.96
VA	161	501904	0.97	0.94	0.95	0.95
VT	12	24662	1.46	1.48	1.47	1.44
WA	82	294895	0.84	0.86	0.85	0.85
WI	69	243109	0.88	0.97	0.91	0.95
WV	64	201951	0.98	0.95	0.97	0.95
WY	9	25240	1.02	1.04	1.05	1.00

© 2023 Lehr CJ et al. *JAMA Network Open.*

eTable 5. Donor and Recipient Characteristics by US Census Region

ADI quintiles (1 = highest ADI, 5 = lowest ADI), Diagnosis groups (group A, obstructive lung disease; group B, pulmonary vascular disease; group C, cystic fibrosis and immunodeficiency disorders; and group D, restrictive lung diseases).

**Characteristic**	**Midwest N = 4,262**[1]	**Northeast N = 3,973**[1]	**South N = 7,430**[1]	**West N = 3,839**[1]
Candidate Age at Listing	60 (52, 65)	61 (52, 66)	60 (50, 66)	60 (51, 66)
Race and Ethnicity				
Non-Hispanic White	3,666 (86%)	3,177 (80%)	5,744 (77%)	2,788 (73%)
Non-Hispanic Black	373 (8.8%)	374 (9.4%)	934 (13%)	180 (4.7%)
Hispanic	137 (3.2%)	295 (7.4%)	628 (8.5%)	656 (17%)
Asian or Pacific Islander	66 (1.5%)	112 (2.8%)	98 (1.3%)	166 (4.3%)
American Indian or Alaska Native	14 (0.3%)	6 (0.2%)	12 (0.2%)	31 (0.8%)
Multiracial	6 (0.1%)	9 (0.2%)	14 (0.2%)	18 (0.5%)
Recipient ADI Quintile				
1	1,006 (24%)	1,736 (44%)	1,789 (24%)	1,591 (41%)
2	940 (22%)	788 (20%)	1,338 (18%)	676 (18%)
3	991 (23%)	612 (15%)	1,417 (19%)	568 (15%)
4	772 (18%)	415 (10%)	1,508 (20%)	556 (14%)
5	553 (13%)	422 (11%)	1,378 (19%)	448 (12%)
Diagnosis				
A	1,237 (29%)	951 (24%)	1,798 (24%)	897 (23%)
B	185 (4.3%)	219 (5.5%)	267 (3.6%)	191 (5.0%)
C	409 (9.6%)	362 (9.1%)	728 (9.8%)	309 (8.0%)
D	2,431 (57%)	2,441 (61%)	4,637 (62%)	2,442 (64%)
Blood Type				
A	1,734 (41%)	1,574 (40%)	2,891 (39%)	1,457 (38%)
AB	174 (4.1%)	167 (4.2%)	282 (3.8%)	152 (4.0%)
B	468 (11%)	505 (13%)	831 (11%)	385 (10%)
O	1,886 (44%)	1,727 (43%)	3,426 (46%)	1,845 (48%)
BMI	26.1 (22.0, 29.1)	26.3 (22.3, 29.8)	25.9 (22.2, 29.1)	25.7 (21.9, 29.0)
Unknown	2	3	2	2
Functional Status				
1	189 (4.4%)	389 (9.8%)	430 (5.8%)	622 (16%)
2	3,713 (87%)	3,305 (84%)	6,378 (86%)	2,927 (76%)

© 2023 Lehr CJ et al. *JAMA Network Open.*

Characteristic	Midwest N = 4,262[1]	Northeast N = 3,973[1]	South N = 7,430[1]	West N = 3,839[1]
3	350 (8.2%)	263 (6.6%)	592 (8.0%)	286 (7.5%)
Unknown	10	16	30	4
Six-min walk distance	880 (575, 1,128)	800 (518, 1,082)	850 (530, 1,108)	780 (393, 1,094)
Unknown	78	63	106	114
Cardiac Index	2.71 (2.35, 3.20)	2.79 (2.40, 3.26)	2.80 (2.42, 3.29)	2.84 (2.44, 3.35)
Unknown	1,074	1,194	1,699	1,094
Bilirubin	0.40 (0.30, 0.60)	0.40 (0.30, 0.60)	0.50 (0.30, 0.70)	0.50 (0.30, 0.70)
Unknown	1,264	1,085	2,452	1,178
Creatinine	0.81 (0.66, 0.97)	0.81 (0.69, 0.98)	0.80 (0.70, 1.00)	0.80 (0.67, 0.94)
Unknown	22	26	19	18
Supplemental Oxygen (L)	4.0 (2.0, 6.0)	4.0 (3.0, 6.0)	4.0 (3.0, 6.0)	4.0 (3.0, 6.0)
Unknown	584	517	1,048	517
Respiratory Support				
BiPap	344 (8.1%)	289 (7.3%)	513 (6.9%)	250 (6.5%)
Mechanical	229 (5.4%)	191 (4.8%)	428 (5.8%)	252 (6.6%)
None	3,428 (81%)	3,238 (82%)	6,071 (82%)	3,133 (82%)
CPAP	257 (6.0%)	243 (6.1%)	404 (5.4%)	201 (5.2%)
Unknown	4	12	14	3
PCO2	46 (40, 54)	44 (39, 53)	44 (39, 51)	44 (39, 51)
Unknown	174	236	347	92
Supplemental O2 Percent	81 (55, 100)	100 (60, 100)	88 (53, 100)	80 (50, 100)
Unknown	3,768	3,573	6,585	3,431
Pulmonary Artery Systolic Pressure	40 (33, 50)	39 (32, 50)	38 (32, 48)	38 (31, 48)
Unknown	915	1,142	1,397	882
Donor Age	33 (23, 47)	34 (24, 47)	32 (23, 45)	34 (23, 47)
Donor Race				
Non-Hispanic White	2,969 (70%)	2,655 (67%)	4,375 (59%)	1,936 (50%)
Non-Hispanic Black	823 (19%)	738 (19%)	1,762 (24%)	344 (9.0%)
Hispanic	346 (8.1%)	449 (11%)	1,084 (15%)	1,238 (32%)
Asian or Pacific Islander	100 (2.3%)	109 (2.7%)	172 (2.3%)	254 (6.6%)
American Indian or Alaska Native	19 (0.4%)	9 (0.2%)	28 (0.4%)	43 (1.1%)
Multiracial	5 (0.1%)	13 (0.3%)	9 (0.1%)	24 (0.6%)

© 2023 Lehr CJ et al. *JAMA Network Open.*

Characteristic	Midwest<br>N = 4,262[1]	Northeast<br>N = 3,973[1]	South<br>N = 7,430[1]	West<br>N = 3,839[1]
Donor ADI Quintile				
1	800 (19%)	1,049 (26%)	1,269 (17%)	1,060 (28%)
2	733 (17%)	698 (18%)	1,186 (16%)	638 (17%)
3	893 (21%)	709 (18%)	1,334 (18%)	570 (15%)
4	852 (20%)	715 (18%)	1,633 (22%)	668 (17%)
5	984 (23%)	802 (20%)	2,008 (27%)	903 (24%)
Donor Region				
Midwest	2,959 (69%)	747 (19%)	905 (12%)	142 (3.7%)
Northeast	160 (3.8%)	1,947 (49%)	381 (5.1%)	18 (0.5%)
South	986 (23%)	1,202 (30%)	5,918 (80%)	270 (7.0%)
West	157 (3.7%)	77 (1.9%)	226 (3.0%)	3,409 (89%)

[1]Median (IQR); n (%)

© 2023 Lehr CJ et al. *JAMA Network Open.*

eReferences

1. Valeri L, VanderWeele TJ. Mediation analysis allowing for exposure–mediator interactions and causal interpretation: theoretical assumptions and implementation with SAS and SPSS macros. *Psychological methods*. 2013 Jun;18(2):137.

© 2023 Lehr CJ et al. *JAMA Network Open.*
