# Supplementary material for: Association of Socioeconomic Position With Racial and Ethnic Disparities in Survival After Lung Transplant
Source: JAMA Netw Open. 2023 Apr 19;6(4):e238306. doi: 10.1001/jamanetworkopen.2023.8306 (PMC10116361; doi:10.1001/jamanetworkopen.2023.8306)
Supplement: Supplement 2. — Data Sharing Statement [file jamanetwopen-e238306-s002.pdf]

Data Sharing Statement

Lehr. Association of Socioeconomic Position With Racial and Ethnic Disparities in Survival After Lung Transplant. *JAMA Netw Open*. Published April 19, 2023. doi:10.1001/jamanetworkopen.2023.8306

Data
Data available: No

Additional Information
Explanation for why data not available: Data is publicly available through the Scientific Registry of Transplant Recipients.
